# Impact of the COVID-19 pandemic on CTSA Clinical Research Centers over 2 years

**DOI:** 10.1017/cts.2023.543

**Published:** 2023-05-10

**Authors:** Mary H. Samuels, Ella Hommeyer, Brian Booty, Kelli Frost, Cynthia Morris

**Affiliations:** Oregon Clinical and Translational Research Institute, Oregon Health & Science University, Portland, OR, USA

**Keywords:** Clinical Research Centers, COVID-19 pandemic, CTSA hubs, survey, patient-oriented research

## Abstract

**Introduction:**

The COVID-19 pandemic had an abrupt impact on patient-oriented research early in the pandemic. CTSA Clinical Research Centers (CRCs) rapidly adapted to this challenge, but the continued impact of later phases of the pandemic on CRC operations is not clear.

**Methods:**

An online REDCap survey of CTSA CRCs was developed that covered the first 2 years of the pandemic. The survey focused on impact on CRC functions, mitigation strategies, recovery of CRC activities, CRC contributions to COVID-related research, and potential lessons for future public health emergencies. The survey was sent to CRC directors at 61 CTSA Hubs in May 2022.

**Results:**

Twenty-seven Hubs (44%) responded to the survey. Most CRCs reported greater than 50% declines in inpatient census in the first year of the pandemic, with less severe impacts on outpatient census. CRCs pivoted to support COVID-related research and adopted innovative technology-driven approaches to support clinical research. Census improved in the second year of the pandemic in most CRCs but often remained below pre-pandemic levels, and greater than half of CRCs reported decreased revenue.

**Conclusions:**

CTSA-supported CRCs faced unprecedented challenges at the onset of the COVID-19 pandemic and responded rapidly to support COVID-related research and implement innovative approaches that allowed patient-oriented research activities to resume. However, many CRCs continued to report decreased research activities in the second year of the pandemic, and the long-term effects on CRC operations on finances are not clear. CRCs will likely need to evolve to provide support in nontraditional ways.

## Introduction

The COVID-19 global pandemic created unprecedented challenges for the biomedical research enterprise, as safety concerns led to the temporary cessation of most clinical research activity, and staff and resources redeployed to clinical care and COVID-related research. As safety protocols were implemented for staff and patients and highly effective vaccines were introduced, clinical research activity has gradually resumed, but it is not clear whether pre-pandemic levels of activity have been regained.

The Clinical Research Centers (CRCs) within the national Clinical and Translational Science Award (CTSA) consortium were vital for the conduct of patient-oriented research prior to the pandemic and played a major role to support COVID-related research as the pandemic unfolded. However, there were widespread interruptions in non-COVID-related clinical research activities during this time. CTSA-wide surveys have been conducted that describe the challenges and adaptations among CRCs during the early phase of the pandemic [1–5], but none have been conducted more recently than the fall of 2020. To understand the impact of later phases of the pandemic on CRC functioning, we conducted an online survey of CTSA CRCs that covered the first 2 years of the pandemic, through February of 2022. We focused on the impacts on overall CRC functions, successful strategies to mitigate negative impacts, recovery of CRC activities over time, contributions of CRCs to COVID-related research, and potential lessons for future public health emergencies as CRCs navigated the second year of the pandemic.

## Materials and Methods

An online REDCap survey was developed by the authors, with questions pertaining to CTSA CRC functioning during the first 2 years of the COVID-19 pandemic. The survey was divided into two main sets of questions; the first set focused on non-COVID-related research activity and also included questions related to the impact of the pandemic on CRC financial status, research staff, and early career investigators. The second set of questions focused exclusively on COVID-related research activity. The questions in each section were divided into two time periods: the first year of the pandemic (March 2020–Feb 2021) and the second year (March 2021–Feb 2022). Questions were designed for both quantitative and qualitative replies. The survey was reviewed by the Oregon Health & Science University Institutional Review Board (IRB), which determined that the survey process did not require IRB approval. The survey is provided in the appendix as Supplementary Material.

An email was sent to the 61 CTSA Hubs listed on the NCATS CTSA website on May 24, 2022. Contact emails for directors or managers of each Hub’s CRC were obtained from Hub websites. If a director or manager could not be identified from the website, the email was sent to the Hub’s principle investigator with a request to forward the email to the appropriate senior staff person. The email contained a brief description of the purpose of the REDCap survey and a link to the survey. The email informed survey participants that all responses were anonymous. Two follow-up email requests to complete the survey were sent to Hubs that had not replied to earlier requests on June 2, 2022 and July 7, 2022.

## Results

Twenty-seven Hubs (44%) from 23 states replied to the survey. One Hub responded from each of 20 states, while 3 states included more than one Hub. Hubs were located in the Northeast (*N* = 8), Midwest (*N* = 10), Southern (*N* = 3), and Western (*N* = 6) regions of the USA. Quantitative responses were collated as percentages of respondents that indicated each response. Narrative comments were grouped together based on common themes. For clarity, survey questions and responses are presented here under three main categories: impact of the pandemic on non-COVID-related CRC research activities (first section of the survey); impact on CRC financial status, research staff, and early career investigators (first section of the survey); and CRC participation in COVID-related research (second section of the survey).

### Impact of the COVID-19 Pandemic on Non-COVID-Related CRC Research (Figs. 1 and 2)

The first set of survey questions referred to clinical research activity that was not related to the COVID pandemic, that is, excluding COVID diagnostic, vaccine, or therapeutic trials. We asked respondents to quantify the effect of the pandemic on non-COVID-related inpatient or outpatient census separately during the first versus the second years of the pandemic. We also asked them to provide narrative reasons for the effects of the pandemic on non-COVID-related research census and how they were able to mitigate these effects.

**Figure 1.**
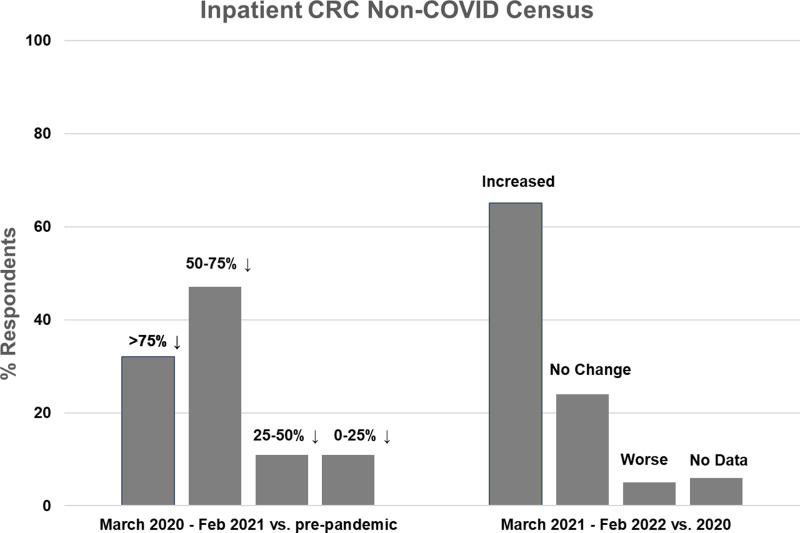
Inpatient non-COVID Clinical Research Center (CRC) census during the first year of the COVID-19 pandemic (left side) and recovery during the second year compared to the first year (right side).

**Figure 2.**
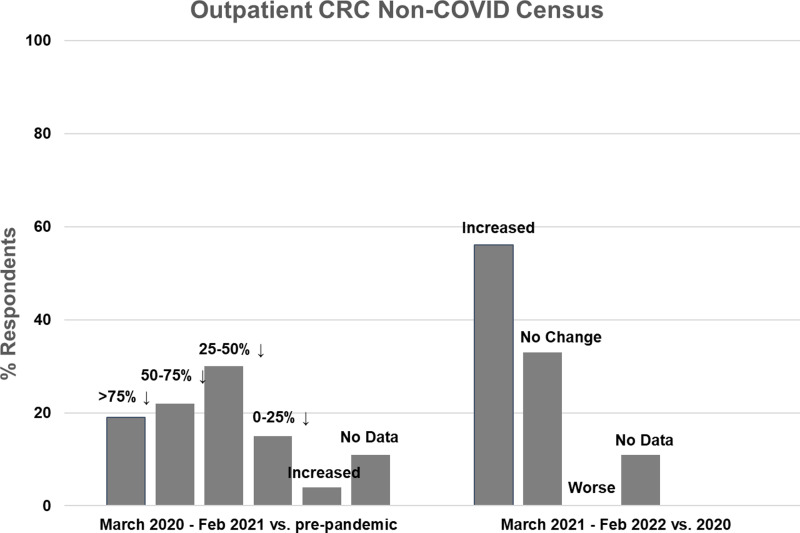
Outpatient non-COVID Clinical Research Center (CRC) census during the first year of the COVID-19 pandemic (left side) and recovery during the second year compared to the first year (right side).

Nineteen CRCs (70% of respondents) reported that they had an inpatient unit. Of those, 17 (89%) reported decreased inpatient census during the first year of the pandemic, compared to pre-pandemic levels. The majority of CRCs (*n* = 15, 79%) experienced at least a 50% decline in inpatient census, with 6 (32%) reporting a greater than 75% decline and 9 (47%) reporting a 50–75% decline. Two CRCs (11%) experienced a 25–50% decline and 2 (11%) experienced less than a 25% decline (Fig. 1, left). Compared to the first year of the pandemic, inpatient census improved in 13 CRCs (68%) during the second year, was stable in 4 (21%), and decreased further in 1 (5%) (Fig. 1, right). Inpatient census had not fully recovered to pre-pandemic levels in 11 CRCs (58%) during the second year of the pandemic.

Twenty seven CRCs (100%) reported that they had one or more outpatient units. Of those, 19 (70%) reported decreased outpatient census during the first year of the pandemic, compared to pre-pandemic levels. Five CRCs (19%) experienced a greater than 75% decline in outpatient census, 6 (22%) experienced a 50–75% decline, 8 (30%) experienced a 25–50% decline, and 4 (15%) experienced a 0–25% decline (Fig. 2, left). One CRC (4%) reported increased outpatient census. Compared to the first year of the pandemic, outpatient census improved in 15 CRCs (56%) during the second year, was stable in 9 (33%), and did not decrease further in any CRC (Fig. 2, right). Outpatient census had not fully recovered to pre-pandemic levels in 10 CRCs (37%) during the second year of the pandemic.

Nineteen CRCs provided narrative responses regarding reasons for decreased research census, many listing multiple reasons:

- Thirteen CRCs (68% of respondents to this question) reported COVID-related safety concerns from investigators, staff, and/or participants.
- Eight CRCs (42%) reported institutional, state, federal, and/or industry sponsor restrictions.
- Seven CRCs (37%) reported staffing shortages due to RN’s leaving or being reassigned to COVID-related research or hospital needs.
- Four CRCs (21%) reported transitions to virtual research visits, eConsent, and mail delivery of research medications to participants.
- Three CRCs (16%) reported restrictions on visits due to the need for COVID testing or social distancing requirements, or canceling visits due to symptoms or exposures.
- Reasons listed by one or two CRCs included loss of principle investigators, investigators not restarting protocols that had been halted, inability to see immunocompromised patients, space and staff limitations due to reallocation by the hospital, or diversion of resources to COVID vaccine trials.

Nineteen CRCs reported mitigation strategies for the decreased census:

- Nine CRCs (47% of respondents to this question) pivoted to support COVID testing and research studies.
- Five CRCs (26%) emphasized close coordination and frequent clear communication with their institution, investigators, and research participants, especially regarding safety protocols.
- Four CRCs (21%) modified protocols to decrease in-person visits in order to keep research protocols open.
- Three CRCs (16%) implemented staffing modifications, including redeploying staff to other areas, agency nursing, and overtime.

### Impact of the COVID-10 Pandemic on CRC Financial Status, Research Staff, and Early Career Investigators (Figs. 3 and 4)

Additional questions in the first section of the survey asked about the impact of the pandemic on CRC financial status, research staff, and early career investigators.

**Figure 3.**
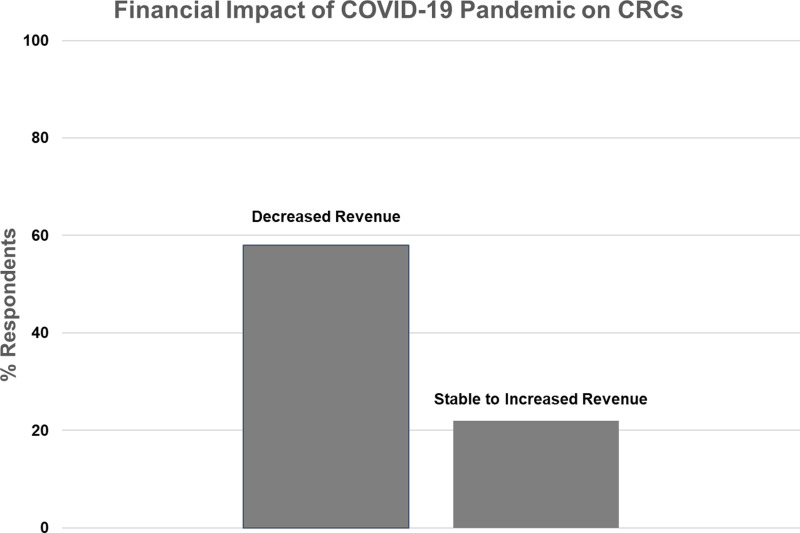
Financial impact of the COVID-19 pandemic on Clinical Research Centers (CRCs).

**Figure 4.**
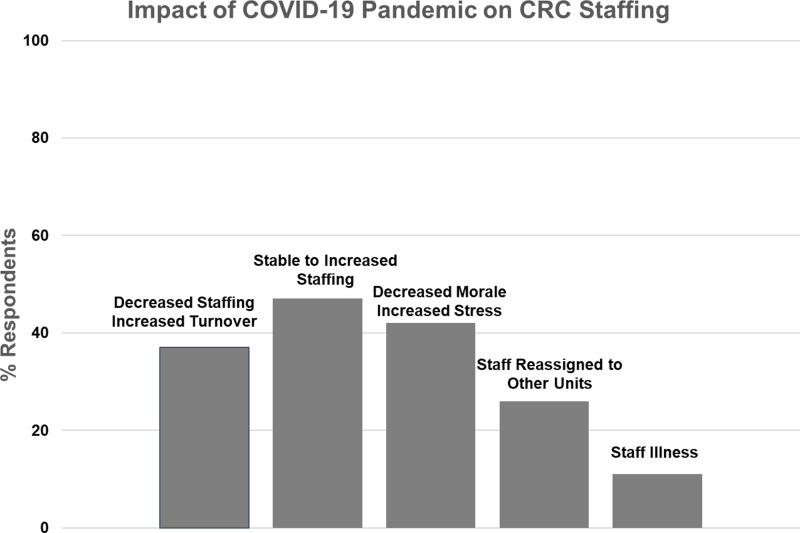
Impact of COVID-19 on Clinical Research Center (CRC) staffing.

Nineteen CRCs (70%) responded to the question regarding their financial status during the pandemic (Fig. 3). Eleven (58%) reported decreased overall revenue during the pandemic, while eight (42%) reported stable or increased revenue. In the latter case, four CRCs specifically mentioned increased revenue from COVID-related research studies, one CRC moved coordinators to other projects to maintain revenue, and one mentioned that studies were more intense and generated more revenue.

Nineteen CRCs (70%) provided narrative responses to the question regarding pandemic effects on their clinical research staff, in the following categories (Fig. 4):

- Nine CRCs (47%) reported stable to increased staffing, while seven (37%) had decreased staffing and increased turnover.
- Eight CRCs (42%) described that their staff felt overworked and fatigued, with increased stress and decreased morale, while three reported no change in morale.
- Five CRCs (26%) reported that they had staff reassigned to other hospital functions, and two (11%) were impacted by significant staff illness.

Eighteen CRCs (67%) provided narrative responses to the question regarding pandemic effects on early career investigators. Thirteen (72%) reported that their early career investigators experienced significant challenges due to overall “difficulties functioning,” delays in recruitment and study conduct, tabling of new projects, losing staff, and difficulties meeting grant deadlines to spend funds. In some cases, early career investigators shifted their work to outside the Hub structure or left the institution due to these limitations. Two CRCs reported no problems, and three did not know or had neutral comments.

### CRC Participation in COVID-related Research (Fig. 5)

The second section of survey questions referred to clinical research activity related to the COVID pandemic (i.e., COVID vaccine, noninterventional, or therapeutic trials). We asked respondents to state whether they were involved with each type of COVID-related research and to describe the type of activities they supported and resources they provided.

**Figure 5.**
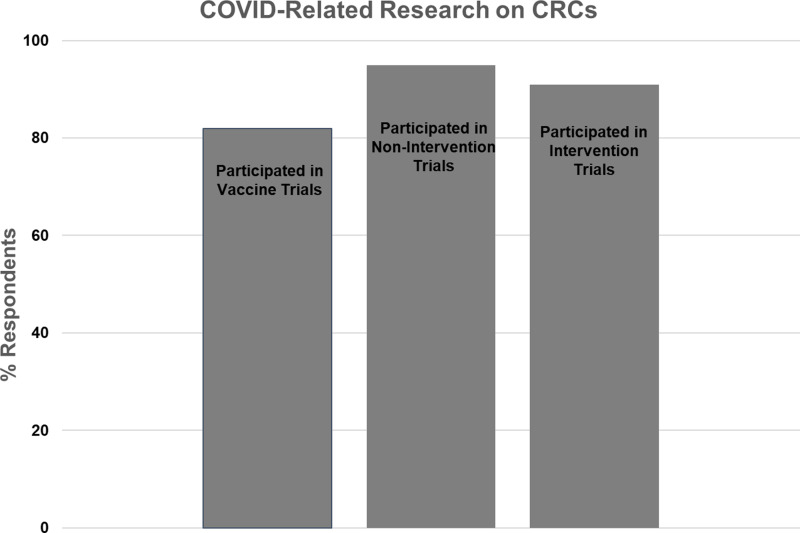
Participation of Clinical Research Centers (CRCs) in COVID-related research.

Twenty-two CRCs (81%) responded to the question regarding COVID vaccine trials, with 18 (82%) reporting involvement and 4 (18%) reporting no involvement. The majority of CRCs reported assisting with study start-up, study visits, regulatory issues, budget management, and/or the research pharmacy. One CRC reported establishing a repository for COVID vaccine trial biospecimens.

Twenty-two CRCs (81%) responded to the question regarding COVID noninterventional studies (e.g., blood sampling for assay development and monitoring of immune response). Twenty one (95%) reported involvement, and one (5%) reported no involvement. The majority of CRCs reported that they conducted study visits and handled samples. One CRC led strategies for eConsent and virtual and home visits, while another CRC provided access to retrospective and prospective data and biospecimens.

Twenty-two CRCs (81%) responded to the question regarding COVID treatment trials, with 20 (91%) reporting involvement and 2 (9%) reporting no involvement. The majority of CRCs reported that they provided nursing, regulatory, and/or pharmacy support for inpatient and/or outpatient trials.

Twenty-two CRCs (81%) responded to the question regarding the proportion of overall research efforts that were directed to all types of COVID-related research. One CRC (5%) reported that over 75% of their effort was related to COVID studies, 4 (18%) reported 50–75% effort, 12 (55%) reported 25–50% effort, and 5 (23%) reported 0–25% effort.

Seventeen CRCs (63%) responded to questions regarding whether COVID-related research interfered with non-COVID research activities. Five CRCs (29%) reported that this occurred. Reasons included scheduling difficulties for rooms and staff due to high-volume enrollment for COVID studies, with competition for limited resources. In contrast, 12 CRCs (71%) reported that COVID-related research efforts did not interfere with non-COVID-related research. Narrative responses stated that COVID-related research helped offset decreases in non-COVID research, in one case allowing the CRC to hire additional staff. Two CRCs reported that COVID studies first helped mitigate decreased activity levels, but then interfered with usual activities as non-COVID studies resumed. One CRC pointed out that balancing COVID and non-COVID studies required innovative staffing models, more reliance on non-RN research personnel, and efficiencies in training new staff.

## Discussion

Our survey results show that there was a major negative impact on CTSA Hub CRC activity during the first year of the COVID-19 pandemic, with 79% of responding CRCs with inpatient units reporting greater than 50% declines in inpatient census and 32% reporting greater than 75% declines. There was also a negative impact on outpatient census, but the effect appeared to be less severe. The reasons for this are not entirely clear, but perhaps staffing constraints were less significant for outpatient research visits, which are shorter in duration, do not necessarily require RN staffing, and tend to be less staff-intensive. Given the widespread constraints on nontherapeutic clinical research activity early in the pandemic, it is highly likely that the remaining admissions and outpatient research visits were largely for therapeutic trials [3]. Our survey did not include questions regarding specific patient populations (e.g., elderly, immunocompromised, and cancer patients), but a few of the narrative responses indicated that investigators were particularly reluctant to conduct in-person visits for those vulnerable research participants if there were any other options.

These data confirm earlier institutional and CTSA-wide surveys and are not surprising, given the widespread mandated restrictions on non-COVID-related clinical research early in the pandemic [2–4,6–8]. Additional common reasons for decreased activity reported in this and previous surveys include concerns for the safety of research participants, investigators, and staff, and staffing shortages.

Previous CTSA-wide surveys of CRCs were conducted in 2020, during the first year of the pandemic, when less was understood regarding COVID-19 transmission, personal protective equipment was limited, and COVID-19 vaccines had not yet been developed [1–4,6,7]. Our survey is the first one that covers the second year of the pandemic, adding valuable insights to how CRCs started to recover from initial severe constraints on activities. We found that inpatient and outpatient research census improved from the first to the second year in most CRCs. However, census often remained below pre-pandemic levels, despite the easing of most institutional restrictions on clinical research activities. Reasons for this included continued safety concerns from participants and investigators and staffing shortages. Many CRCs reported that they mitigated the negative impact on research activities by pivoting to support COVID-related research, maintaining close coordination with institutional entities and investigators, implementing practices to mitigate risks to research personnel, and adopting innovative approaches to supporting clinical research during the pandemic, including eConsent, virtual visits, electronic source documentation, and home delivery of experimental therapeutic agents [1–4,9–11]. These latter approaches allowed outpatient clinical research to resume, but most would not be captured as CRC census.

Greater than half of the CRCs responding to the survey reported decreased revenue during the pandemic. CRCs are prohibited from funding direct research activities with CTSA award monies and rely heavily on revenue from investigator grants and contracts, as well as institutional support. For some CRCs, loss of revenue from non-COVID-related research activities was mitigated by increased revenue from COVID-related research. However, as COVID-related clinical research wanes and if non-COVID research census does not return to pre-pandemic levels, there may be long-term implications for CRC operating budgets.

Approximately half of CRCs were able to maintain staffing levels during the 2 years of the pandemic covered in this survey, but there were frequent reports of decreased staffing levels, increased turnover, and low morale, similar to previous reports from earlier in the pandemic [3]. These reports echo more widespread concerns regarding long-term effects of the pandemic on the nursing workforce.

CRCs have been major sites of support for early career investigators who conduct patient-oriented clinical research. The majority of CRCs responding to our survey reported that their early career investigators experienced significant challenges in continuing their research, confirming earlier reports [12–14]. It is hoped that these challenges will abate, but longer-term effects of the pandemic on career development should be monitored, with consideration of strategies to reverse this impact.

Our survey provided unique data regarding participation of CRCs in COVID-related research and its impact on non-COVID activities. The great majority of CRCs responding to our survey participated in COVID-related research efforts, including vaccine trials, noninterventional studies (e.g., biorepositories, serology studies, and diagnostic development), and therapeutic trials. CRC support spanned the spectrum of clinical research activities, including study start-up, regulatory support, training of clinical research staff, and conduct of study visits, confirming earlier reports [5,11]. Participation in noninterventional studies was higher than in therapeutic trials, likely representing lower staffing barriers to conducting noninterventional studies. The proportion of overall effort devoted to COVID-related studies varied widely, which may reflect variability in patient numbers, staff availability, and resources that could be quickly deployed for large-scale vaccine trials or studies involving actively infected COVID-19 patients. It is a tribute to the robust CTSA CRC infrastructure that this pivot to COVID-related research was largely successful. In most cases, this did not interfere with non-COVID research and in fact helped maintain research census, although this was during a time when non-COVID research was constrained.

Despite the valuable information derived from our survey, especially regarding efforts during the second year of the pandemic, there are limitations to our data. Our response rate of 44% was lower than previous CTSA surveys and may limit the generalizability of our results. The geographic diversity of responses mitigates this limitation but does not eliminate it. In particular, there was relative underrepresentation among CTSA Hubs in Southern states, with 20% of Southern Hubs responding, compared to 43–67% of Hubs from other US regions. Given that some Southern states’ populations were disproportionately affected by the pandemic, this limits our ability to assess whether there was a differential impact of the pandemic on CRCs depending on region. In an effort to minimize the time commitment to complete the survey, we asked respondents to provide estimated answers to quantitative questions, rather than calculating exact numbers from their internal databases. This may have led to under- or overestimating numerical data. The broad range of possible responses to our quantitative questions did not allow for correlative analyses of potential relationships between changes in census and changes in revenue, staffing turnover or decreased workforce morale. Fewer respondents provided comments for some qualitative questions, limiting generalizability of these issues.

A number of publications from CTSA sites have outlined lessons learned from the COVID-19 pandemic and proposals to prepare the clinical research enterprise for future public health emergencies [1–4,6,15]. Our survey results underscore many of these findings, highlighting the value of a robust patient-oriented clinical research infrastructure that can rapidly respond to evolving events by reducing nonessential research activities, pivoting to innovative methods that allow clinical research to proceed in a virtual environment, and deploying CRCs’ considerable resources to support pandemic-related research [16]. Previous publications have highlighted the additional role that CRCs and CTSA Hubs can play in prioritizing clinical research studies for enrollment of patients into COVID-related trials, utilizing fair and transparent criteria that include direct benefit to patients, avoidance of competition for enrollment, protection of providers, efficient use of limited resources, health disparities, and access to care [1,11,16].

While these are positive results, our survey also highlights the challenges that face CRCs in a rapidly evolving public health emergency and its aftermath. Many of the innovative approaches to conducting clinical research are likely to become permanent, as the pandemic hastened technology-driven solutions such as eConsent and remote study visits, and CRCs will need to evolve to provide support in nontraditional ways [2,4,8]. The current financial structure that supports CRC operating budgets may need to evolve as well, as on-site research admissions and visits may not always recover to pre-pandemic levels, and future clinical research growth is likely to incorporate these new approaches. Concerns regarding nursing staffing remain, as staff burn-out and turnover continues to constrain clinical as well as research activities across the country. Long-term solutions for the nursing staffing crisis are not yet clear, but CRCs can encourage staff retention and morale by supporting staff training in research methods and highlighting the unique rewards of clinical research, particularly research conducted at the local level. Finally, sharing lessons learned and plans for future readiness across the CTSA network of CRCs can be invaluable for avoiding pitfalls and highlighting successful operations during a time of unprecedented challenges. Previous CTSA publications have recommended that institutions complete readiness evaluations and put in place workflows, training, and liaison services that can be rapidly deployed for future public health emergencies [4,6,7,15,17].

## Supplementary material

For supplementary material accompanying this paper visit https://doi.org/10.1017/cts.2023.543. click here to view supplementary material
