# Supplementary material for: Impact of the COVID-19 pandemic on CTSA Clinical Research Centers over 2 years
Source: J Clin Transl Sci. 2023 May 10;7(1):e116. doi: 10.1017/cts.2023.543 (PMC10225268; doi:10.1017/cts.2023.543)
Supplement: Supplementary file 1 [file S2059866123005435sup001.docx]

**Survey of CTSA Clinical Research Centers regarding impact of COVID pandemic**

Thank you for completing this survey, which is designed to assess effects of the COVID-19 pandemic on the functioning and success of the CTSA’s clinical research centers (CRCs). We hope to collate and analyze these data, and share them with the CTSA consortium for lessons learned and strategies for successful operations during the pandemic.

For this survey, “census” refers to the numbers of research participants, admissions, and/or outpatient visits. “Effect” generally refers to the percentage change from pre-pandemic levels of activity, but feel free to provide qualitative responses as well.

The first set of questions refers to clinical research activity that does not include research related to the COVID pandemic (ex. COVID vaccine trials, COVID diagnostic or treatment trials).

1. Does your CRC include an inpatient (overnight stay) unit?

Yes

No

1. If yes, can you estimate the effect of the first year of the pandemic (March 2020-Feb 2021) on your inpatient (non-COVID) research census, compared to pre-pandemic inpatient research census? If possible, please provide an approximate percent change from pre-pandemic levels. Add comments to the text box.

(Five choices here with check boxes: 0-25% decrease, 25-50% decrease, 50-75% decrease, > 75% decrease, other)

Text box for other comments

1. Can you estimate the effect of the second year of the pandemic (March 2021-Feb 2022) on your inpatient (non-COVID) research census, compared to pre-pandemic levels and compared to the first year of the pandemic? If possible, please provide an approximate percent change from pre-pandemic levels. Add comments to the text box.

(Five choices here with check boxes: 0-25% decrease, 25-50% decrease, 50-75% decrease, > 75% decrease, other)

Text box for other comments

1. Does your CRC include an outpatient unit?

Yes

No

1. If yes, can you estimate the effect of the first year of the pandemic (March 2020-Feb 2021) on your outpatient (non-COVID) research census, compared to pre-pandemic outpatient research census? If possible, please provide an approximate percent change from pre-pandemic levels. Add comments to the text box.

(Five choices here with check boxes: 0-25% decrease, 25-50% decrease, 50-75% decrease, > 75% decrease, other)

Text box for other comments

1. Can you estimate the effect of the second year of the pandemic (March 2021-Feb 2022) on your outpatient (non-COVID) research census, compared to pre-pandemic research census and compared to the first year of the pandemic? If possible, please provide an approximate percent change from pre-pandemic levels. Add comments to the text box.

(Five choices here with check boxes: 0-25% decrease, 25-50% decrease, 50-75% decrease, > 75% decrease, other)

Text box for other comments

1. What were some of the reasons for the effects of the pandemic on your non-COVID related inpatient or outpatient research census (patient safety concerns, nursing staffing shortages, etc.)?

Text box for replies

1. Were you able to mitigate negative effects on your CRC’s activity levels during the pandemic? If so, what were your strategies for doing so?

Text box for replies

1. How did the pandemic affect your CRC’s financial status?

Text box for replies

1. How did the pandemic affect your clinical research staff? For example, were you able to remain fully staffed? Did turnover increase? How was morale affected?

Text box for replies

1. How did the pandemic affect early career investigators utilizing your research unit?

Text box for replies

The second set of questions refers to COVID-related clinical research activity on your CRC during the pandemic (ex. COVID vaccine trials, inpatient or outpatient COVID treatment trials, etc.).

1. Did your CRC participate in any COVID related vaccine trials?

Yes

No

1. If yes, can you describe your involvement? What resources did you provide for the vaccine trials? How many participants enrolled?

Text box for replies

1. Did your CRC participate in any COVID related non-interventional research studies (ex. blood sampling for assay development, monitoring of immune response, etc.)?

Yes

No

1. If yes, can you describe your involvement? What resources did you provide for the non-interventional studies? How many participants enrolled?

Text box for replies

1. Did your CRC participate in any COVID related treatment trials?

Yes

No

1. If yes, can you describe your involvement? What resources did you provide for the interventional trials? How many participants enrolled?

Text box for replies

1. Can you estimate what proportion of your research efforts during the past two years of the pandemic (March 2020 – February 2022) was related to COVID related research vs. non-COVID related research? If possible, please provide an estimate or approximate percentage.

Text box for replies

1. Did your COVID related research efforts interfere with non-COVID related research efforts? Did your COVID related research efforts help mitigate decreased non-COVID related efforts?

Text box for replies

Do you have any other comments regarding the pandemic’s effect on your CRC’s functions?

Text box for replies

If you would be willing to discuss these issues with Dr. Samuels in more detail, please list your name and contact information here, and thank you for your input.

Text box here
